# Methylation in MIRLET7A3 Gene Induces the Expression of IGF-II and Its mRNA Binding Proteins IGF2BP-2 and 3 in Hepatocellular Carcinoma

**DOI:** 10.3389/fphys.2018.01918

**Published:** 2019-01-24

**Authors:** Amr A. Waly, Nada El-Ekiaby, Reem A. Assal, Mohamed M. Abdelrahman, Karim A. Hosny, Hend M. El Tayebi, Gamal Esmat, Kai Breuhahn, Ahmed I. Abdelaziz

**Affiliations:** ^1^The Molecular Pathology Research Group, German University in Cairo, Cairo, Egypt; ^2^School of Medicine, Newgiza University, Cairo, Egypt; ^3^Department of General Surgery, Faculty of Medicine, Cairo University, Cairo, Egypt; ^4^Department of Endemic Medicine and Hepatology, Cairo University, Cairo, Egypt; ^5^Molecular Hepatopathology, Institute of Pathology, University Hospital Heidelberg, Heidelberg, Germany

**Keywords:** miR-let-7a, methylation, IGF-II, IGF2BP, HCC

## Abstract

miR-let-7a is a tumor suppressor miRNA with reduced expression in most cancers. Methylation of *MIRLET7A3* gene was reported to be the cause of this suppression in several cancers; however, it was not explicitly investigated in hepatocellular carcinoma (HCC). We aimed at investigating miR-let-7a expression and molecular mode in HCC, identifying drug-targetable networks, which might be affected by its abundance. Our results illustrated a significant repression of miR-let-7a, which correlated with hypermethylation of its gene of origin *MIRLRT7A3*. This was further supported by the induction of miR-let-7a expression upon treatment of HCC cells with a DNA-methyltransferase inhibitor. Using a computational approach, insulin-like growth factor (IGF)-II and IGF-2 mRNA binding proteins (IGF2BP)-2/-3 were identified as potential targets for miR-let-7a that was further confirmed experimentally. Indeed, miR-let-7a mimics diminished IGF-II as well as IGF2BP-2/-3 expression. Direct binding of miR-let-7a to each respective transcript was confirmed using a luciferase reporter assay. In conclusion, this study suggests that DNA hypermethylation leads to epigenetic repression of miR-let-7a in HCC cells, which induces the oncogenic IGF-signaling pathway.

## Introduction

MicroRNA lethal-7 (miR-let-7), has been identified as the second earliest discovered microRNA (miR) in the development of the nematode *Caenorhabditis elegans*, the organism where miRs were first discovered (Reinhart et al., 2000). The naming of let-7 was based on early studies highlighting its crucial role in guiding *C. elegans* differentiation program, and the larval lethality that occurs upon its embryonic inactivation (Rougvie and Ambros, 1995; Slack et al., 2000). In humans, let-7 miRs comprise a family of 13 members and were the first miRs discovered in humans (Roush and Slack, 2008). Let-7 miRs are not expressed in human embryonic stem cells; however, their expression rises gradually upon differentiation and reaches a state of maintained high expression in many adult tissues (Sempere et al., 2004; Schulman et al., 2005). On the contrary, let-7 expression levels drop in various human cancers (Park et al., 2007).

Within the context of cancer, miR-let-7a is mostly regarded as a tumor suppressor miRNA with reduced expression in numerous cancers such as lymphoma, Kaposi sarcoma, lung and ovarian cancers (Takamizawa et al., 2004; Dahiya et al., 2008; O’Hara et al., 2009). Thus, the question on how miR-let-7a expression becomes restricted in cancer was insistently discussed. Of the three genomic origins of the mature miR-let-7a: *MIRLET7A1, MIRLET7A2*, and *MIRLET7A3*, the latter is of special importance owing a well-defined CpG island (Lu et al., 2007). This ∼700 bp long domain in the genomic region *22q12.31* is indicating that miR-let-7a could be epigenetically regulated via DNA methylation. In fact, methylation of MIRLET7A3 gene has been reported in acute myeloid leukemia, ovarian and breast cancer (Lu et al., 2007, 2011; Vrba et al., 2013; Ko et al., 2014). In hepatocellular carcinoma (HCC), induction of miR-let-7a expression was reported to exert a potent tumor-suppressive role both *in vitro* as well as in mouse xenograft models (Liu et al., 2014). Yet, the molecular mechanisms behind the role of miR-let-7a as well as its compromised activity in HCC are not well understood.

Hepatocellular carcinoma is characterized by dysregulation of many oncogenic signaling pathways including the Insulin-like Growth Factor (IGF) pathway. The IGF pathway includes two ligands; IGF-I and IGF-II, where binding of either ligands to the IGF-1 receptor (IGF-1R) stimulates the induction of RAF/MEK/ERK and PI3K/AKT/mTOR signaling pathways leading to increased cell growth, proliferation, survival and migration (Wu and Zhu, 2011). IGF signaling overstimulation in HCC is in part due to abnormally high expression of IGF-II by means of loss of imprinting (Poirier et al., 2003) and correlates with increased HCC cells proliferation (Bae et al., 1998) and tumor neovascularization (Lahm et al., 2002). IGF signaling can be tuned by IGF-II mRNA binding proteins (IGF2BPs) which are reported to influence the fate of IGF-II mRNA (Liao et al., 2011). IGF2BP-2 and 3 are well-established oncogenes whose aberrant expression in HCC leads to excessive cell proliferation and invasion, which culminates in poor prognosis (Jeng et al., 2008; Wachter et al., 2012; Kessler et al., 2013). *In silico* analysis led us to investigate the Insulin-like Growth Factor (IGF) oncogenic pathway as a possible sovereign area for miR-let-7a with IGF-II as well as IGF2BP-2 and 3 as possible downstream targets of miR-let-7a.

In this study we first show that DNA methylation is one possible mode of negative miR-let-7a regulation in HCC cells. Secondly, our data illustrate that miR-let-7a regulates the abundance of tumor-supporting insulin-like growth factor IGF-II through the coordinated regulation of IGF-II itself and probably its stabilizing interaction partners IGF2BP-2/3.

## Patients and Methods

### Patients

Liver tissues were obtained from 16 HCV-induced HCC patients and 9 healthy tissues obtained from liver donors (“healthy controls”) during liver transplantation at the Kasr Al-Ainy Hospital, Cairo University, Egypt. Fresh liver samples were snap frozen by liquid nitrogen and stored at −80°C. All subjects gave their written informed consent and the Cairo University ethical review committee approved the study. The study followed the ethical guidelines of the 1975 Declaration of Helsinki. According to the hospital’s pathology report, 66.6% of the patients had more than one focal lesion. Patients’ clinical parameters are presented in Table 1.

**Table 1:** Clinical parameters of patients.

Age: mean ± SD	49 ± 13.5
Sex: male/female	15/1
Aspartate aminotransferase (AST) (U/l)	100.5 ± 65.8
Alanine aminotransferase (ALT) (U/l)	85.6 ± 95.6
Alkaline phosphatase (U/l)	110.2 ± 60.7
Serum albumin (g/dl)	4.6 ± 1.5
Serum AFP (ng/ml)	155.7 ± 22.3
HCV Ab	100% (16 HCC patients)

### Cell Culture and Genetic Manipulation

Huh7 cells were maintained in Dulbecco’s modified Eagle’s medium (DMEM) (Lonza, Basel, Switzerland) supplemented with 4.5 g/L glucose, 4 mmol/L L-glutamine, 10% fetal bovine serum (Lonza, Verviers, Belgium), 1% Penicillin/Streptomycin/MycoZap (Lonza, Basel, Switzerland) at 37°C in 5% CO_2_. Huh7 cells were transfected with miR-let-7a mimics or antagomirs (MIMAT0000062: Syn-hsa-let-7a-5p miScript miRNA Mimic and MIMAT0000062: Anti-hsa-let-7a-5p miScript miRNA Inhibitor, respectively, Qiagen, Hilden, Germany) according to the manufacturer’s instructions. Briefly, Huh7 cells were seeded in 6-well plates (2.5 × 10^5^ cells/well). At 60% confluence cells were treated with the transfection complex (150 ng oligonucleotides mixed in 6 μl of HiPerFect Transfection Reagent (Qiagen, Hilden, Germany) and 100 μl DMEM). Cells treated with transfection complex lacking oligonucleotides were used as negative controls (mock). Total RNA or proteins were extracted 48 h after transfection. All transfections were done in quadruples and the experiment was done twice for confirmation. For drug-induced DNA demethylation experiments, Huh7 cells were treated with 5 μM decitabine (Sigma-Aldrich, St. Louis, MO, United States) in three biological replicates for 5 days followed by total RNA or genomic DNA extraction.

### Total RNA Extraction and Quantification

Hundred milligram of frozen liver tissue specimens were pulverized. RNA was then extracted using mirVana miRNA Isolation Kit (Ambion, Foster City, CA, United States) according to the manufacturer’s protocol. Huh7 cells were directly lysed in culture plate using Biozol reagent according to the manufacturer’s protocol (Invitrogen, Burlington, ON, Canada). The RNA pellet was washed twice with 75% ethanol then dissolved in DEPC-treated water.

miR-let-7a was reverse transcribed from the extracted RNA samples into complementary DNA using the MicroRNA Reverse Transcription Kit (Applied Biosystems, Foster City, CA, United States). mRNAs of IGF-II, IGF2BP-2/-3 were reverse transcribed using the High-capacity cDNA Reverse Transcription Kit (Applied Biosystems) according to the manufacturer’s instruction. Expression of the housekeeping gene RNU6B was measured and used for miR-let-7a normalization. Beta-2-microglobulin was used for normalization of mRNA expression levels. TaqMan probe-based quantification of gene expression was performed using the following ready-made assays as per the manufacturer’s protocol (hsa-let-7a-5p, 478575_mir; RNU6B, 001093; IGF-II: Hs04188276_m1; IGF2BP2, Hs00538954_g1; IGF2BP3, Hs00559907_g1; and B2M’ Hs00187842_m1), StepOne^TM^ Real-Time PCR instrument (Applied Biosystems) and StepOne^TM^ Software (Applied Biosystems). Gene expression is presented as relative quantitation, which was calculated as follows:

$$\mathrm{Relative quantitation (RQ)}=2^{-\Delta \Delta \text{CT}}$$

where ΔCT is the difference in threshold cycles for target gene and endogenous control and ΔΔCT is the difference in ΔCT for target (patients or Huh7 cells or transfected Huh7 cells) and reference (healthy controls or mock cells).

### Western Blotting

Lysis of cells for protein extraction was done using Cell Lysis buffer (Cell Signaling Technology, Frankfurt, Germany) with PhosSTOP (Roche, Mannheim, Germany) and Protease Inhibitor Cocktail Mix G (Serva, Heidelberg, Germany). Cells were scraped off culture dishes, transferred to 1.5 ml tubes, sonicated 3× for 30 s. followed by 10 min centrifugation at 14,000 rpm, 4°C. Supernatants were then transferred to clean tubes and protein concentrations were measured using Bradford Assay (Bio-Rad, Munich, Germany) according to manufacturer’s instructions.

Forty microgram of total protein lysates were loaded and run on a 10% SDS-polyacrylamide gel and subsequently transferred to nitrocellulose membrane. Membrane was blocked using 5% skim-milk powder in TBST for 1 h followed by incubation with the following primary antibodies at 4°C overnight: mouse anti-IMP-1/2/3 (A2), which detects the three IGF2BP isoforms, namely IGF2BP1, 2, and 3 (anti-IGF2BP antibody, sc-271785, Santa Cruz Biotechnology, Heidelberg, Germany) diluted 1:200 and chicken anti-GAPDH diluted 1:10,000 (AB2302 EMD Millipore, Darmstadt, Germany) in 5% skim-milk powder. Membrane was washed 3× with TBST, incubated with the fluorescent secondary antibodies donkey-anti-mouse and donkey-anti-chicken diluted 1:2,000 in 5% skim-milk powder (IRDye coupled, 800 CW, LI-COR biosciences). Protein bands were quantified by measuring relative fluorescence units using Image Studio^TM^ Lite software. The western blots were performed in two biological and two technical replicates.

### Analysis of DNA Methylation

Genomic DNA was extracted from Huh7 cells, HCC and healthy liver tissues using the QIAamp DNA Mini Kit (Qiagen) according to the manufacturer’s protocol. Methylation-sensitive restriction enzymes coupled with quantitative PCR (MSRE-qPCR) was performed using the OneStep qMethyl^TM^ Kit (Zymo Research, Irvine, CA, United States). In brief, two reaction mixes were prepared for each DNA sample: the test reaction mix and reference reaction mix. Each reaction mix contained 20 ng DNA, 10 μM primers (Supplementary Table 1) and twofold concentrated Test or Reference Reaction PreMix. Reaction tubes were placed in a *StepOne* PCR machine with the following thermal conditions: 2 h at 37°C; 10 min at 5°C; 40 cycles 30 s at 95°C, 1 min 60°C, 1 min at 72°C followed by a melting curve analysis to check specificity of the amplicon. For each sample, the percentage of DNA methylation was calculated using the following formula: Methylation (%) = 100 × 2^−ΔCt^; where ΔCt = Ct(test) – Ct(reference). This test was performed for three independent passages of Huh7, 4 HCC and 4 healthy liver DNA samples in duplicates.

### Luciferase Reporter Assay

Online miRNA target prediction software such as Miranda ^1^, miRDB^2^, RNA hybrid^3^, and miRTarBase^4^ were used for target mRNA prediction (Figure 2). Sense and antisense Oligonucleotides corresponding to the identified target sites were designed in a way that, when annealed, to be flanked by sticky ends resembling those of SacI and XbaI digestion (Supplementary Table 2). Target sites were cloned in the pmirGLO Dual-Luciferase miRNA Target Expression vector (Promega, Madison, WI, United States) downstream of the firefly luciferase gene. In brief, the vector was double digested by SacI and XbaI restriction enzymes (Thermo Scientific, United States) and the sticky ended annealed oligonucleotides were inserted into the vector using T4 DNA ligase with 2 h incubation at 16°C. FuGENE HD transfection reagent (Promega) was used to transfect 0.5 μg of each vector in Huh7 cells alone or in combination with 150 ng of miR-let-7a mimics. Forty-eight hours after transfection, luciferase assay was performed using Dual-Luciferase Reporter Assay System (Promega, Mannheim, Germany) according to manufacturer’s instructions. To test for correctness of insert, the constructs were sequenced (SeqLab, Microsynth, Heidelberg, Germany).

### Statistical Analysis and Software

Data are expressed as mean ± standard error of the mean and were analyzed using unpaired Student *t-*test (GraphPad Prism 5). *P*-values lower than 0.05 were considered statistically significant.

MIRLET7A3 gene sequence was retrieved using UCSC Genome browser^5^ and the CpG islands were identified using the online tool Sequence Manipulation Suite^6^.

This tool identifies potential CpG island regions using the method described by Gardiner-Garden and Frommer (1987). Sequence ranges where the Obs/Exp value is larger than 0.6 and the GC content is more than 50% are defined as CpG islands. The expected number of CpG dimers in a window is calculated as the number of ‘C’s multiplied by the number of ‘G’s in the window, divided by the window length.

## Results

### Low Expression Level of miR-let-7a in HCC Cells Is Regulated by Genomic Hypermethylation

**FIGURE 1:**
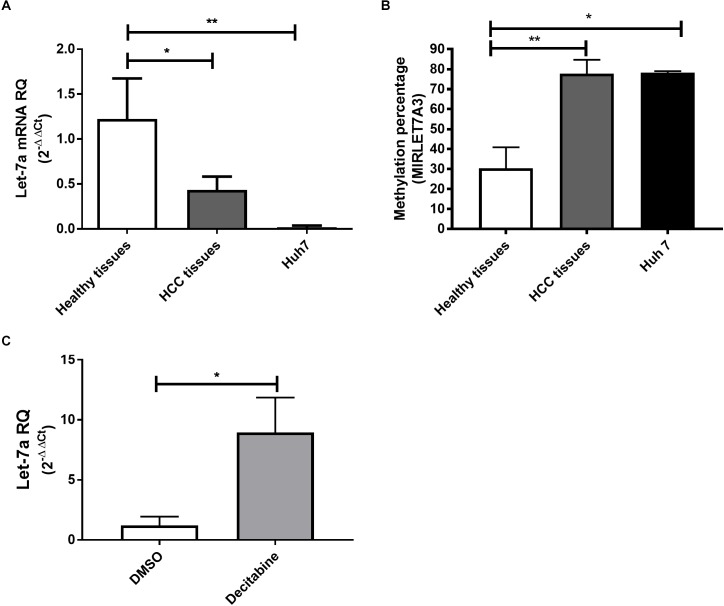
Expression pattern and methylation of miR-let-7a in liver tissues and cell lines. The expression levels of miR-let-7a were found to be significantly down-regulated in HCC tissues (*P* < 0.05) and cell lines (*P* < 0.01) compared to healthy controls **(A)**. MSRE-qPCR analysis revealed significantly higher methylation levels in HCC tissues and Huh7 cell line with average methylation percentage of 77.5% (*P* < 0.01) and 78.15% (*P* < 0.05), respectively, compared to healthy liver tissues 28.2% **(B)**. Treatment of Huh7 cells with decitabine, significantly increased miR-let-7a expression compared to untreated controls (*P* < 0.05) **(C)**. RQ, relative quantitation; ^∗∗^*P* < 0.01; ^∗^*P* < 0.05.

To first compare the expression levels in a human HCC cell line, HCC and healthy liver tissues, qRT-PCR for miR-let-7a was performed (Figure 1A). The analysis showed that in both HCC tissues and HuH7 cells the levels of miR-let-7a were significantly lower compared to healthy liver tissues (*p* = 0.02 and *p* = 0.01, respectively). These results indicate that HuH7 cells may represent a suitable *in vitro* model for further analyses.

After our observation that miR-let-7a is reduced in HCC cells, we decided to investigate the underlying regulatory mechanism for this possible repression. For this, the sequence of *MIRLET7A3* gene (*22q12.31*, 26,753–26,823) located within the *MIRLET7BHG* (46,085,997–46,113,928) was obtained from UCSC Genome Browser and the presence of CpG islands was analyzed using the software CpG Islands-Bioinformatics.org. A well-defined CpG island with about 800 base pairs on chromosome *22q12.31* (26,118–26,919) was detected in the middle of the *MIRLET7A* gene a few base pairs upstream of the sequence of the precursor-miR-let-7a-3 (Supplementary Figure 1). MSRE-qPCR analysis was performed to detect DNA methylation of the identified region in DNA extracted from healthy, HCC tissues and Huh7 cells, which revealed high degree of methylation in HCC tissues with average methylation percentage of 77.5% ranging from 60 to 98.6%. The presence of high methylation levels was also observed in HCC cell lines with a methylation percentage average of 78.15% and a range from 77.3 to 79%. Methylation could be also detected in healthy liver tissues but at much lower levels (average methylation percentage of 28.2% and range from 7 to 56.6%) (Figure 1B). These results would suggest that the miR-let-7a is repressed in HCC cells on the transcriptional level via *MIRLET7A3* gene hypermethylation.

Having first evidence that miR-let-7a levels are low in HCC cells due to epigenetic DNA methylation, we aimed at confirming this molecular mechanism experimentally. Therefore, Huh7 cells were treated with the DNA Methyltransferase (DNMT) inhibitor decitabine followed by assessment of changes in miR-let-7a expression levels. Indeed, decitabine-treatment significantly increased miR-let-7a levels in HCC cells (*p* = 0.028) (Figure 1C).

Together, these results illustrate that genomic hypermethylation of miR-let-7a is one possible mechanism for its reduced expression in HCC cells.

### miR-let-7a Is a Regulator of IGF2BPs

**FIGURE 2:**
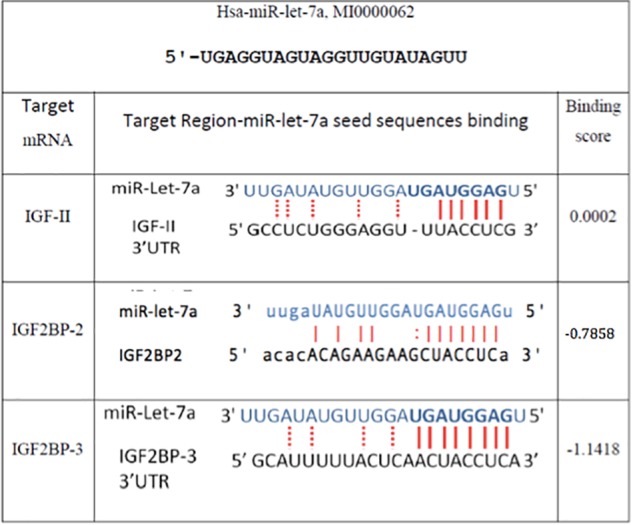
*In silico* prediction of miR-let-7a target genes. Miranda (see text footnote 1), RNA hybrid (see text footnote 3), and Target Scan (www.targetscan.org/) online miR target prediction software suggested that IGF-II and IGF2BP-2 and 3 are putative targets for miR-let-7a. The binding scores illustrated are according to Miranda software.

In order to understand the molecular mechanisms by which miR-let-7a performs its tumor-suppressive function in HCC, we intended to define networks of miR-let-7a targets, which might be involved in the regulation of tumor-relevant pathways. Since we are mainly interested in the IGF signaling pathway and its role in hepatocarcinogenesis (El Tayebi et al., 2011, 2015; Assal et al., 2015; Fawzy et al., 2016; Habashy et al., 2016; Youness et al., 2016; Rahmoon et al., 2017), we selected IGF-axis members potentially targeted by miR-let-7a using *in silico* microRNA target prediction softwares. This analysis yielded IGFII, IGF2BP2 as well as IGF2BP3 as potential miR-let-7a targets (Figure 2).

**FIGURE 3:**
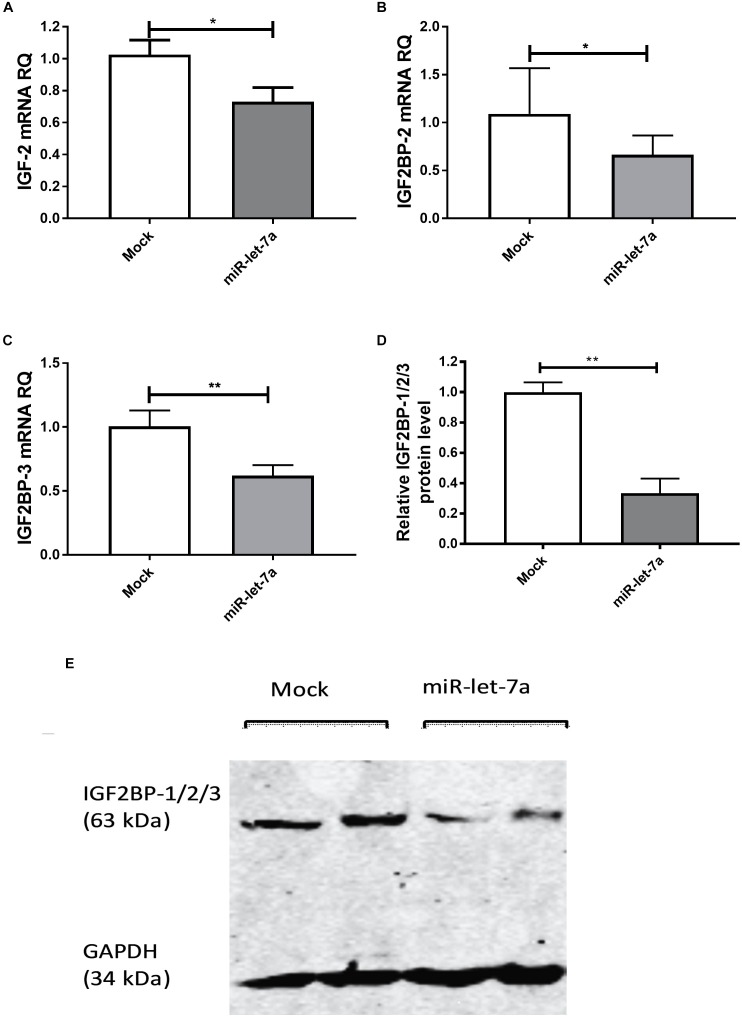
miR-let-7a suppresses IGF-II, IGF2BP-2 and -3. Upon mimicking of miR-let-7a in Huh7 cells, IGF-II mRNA expression was significantly downregulated compared to mock controls (*P* < 0.05) **(A)**. Mimicking of miR-let-7a in Huh7 cells resulted in a significant downregulation of IGF2BP-2 and IGF2BP-3 mRNA (*P* < 0.05 and *P* < 0.01, respectively) **(B,C)** and protein (*P* < 0.01) **(D,E)** levels. RQ, relative quantitation; ^∗∗^*P* < 0.01; ^∗^*P* < 0.05.

To experimentally validate that miR-let-7a could regulate the abundance of these potential target, the expression of miR-let-7a was increased by a respective mimic in Huh7 cells. Transfection with miR-let-7a mimics led to a significant suppression of IGF-II and IGF2BP family transcripts (Figure 3). In detail, miR-let-7a/mimic levels were elevated around 150 fold, which led to a significant downregulation of IGF-II mRNA in Huh7 cells compared to mock controls (*p* = 0.0429) (Figure 3A). Transfection of miR-let-7a mimics also resulted in a significant down regulation of IGF2BP-2 and -3 on the mRNA level (*p* = 0.0042 and *p* = 0.003, respectively) (Figures 3B,C). In addition, western immunoblotting analyses confirmed a negative effect of miR-let-7a on IGF2BP family members (Figures 3D,E) (*p* = 0.0012).

**FIGURE 4:**
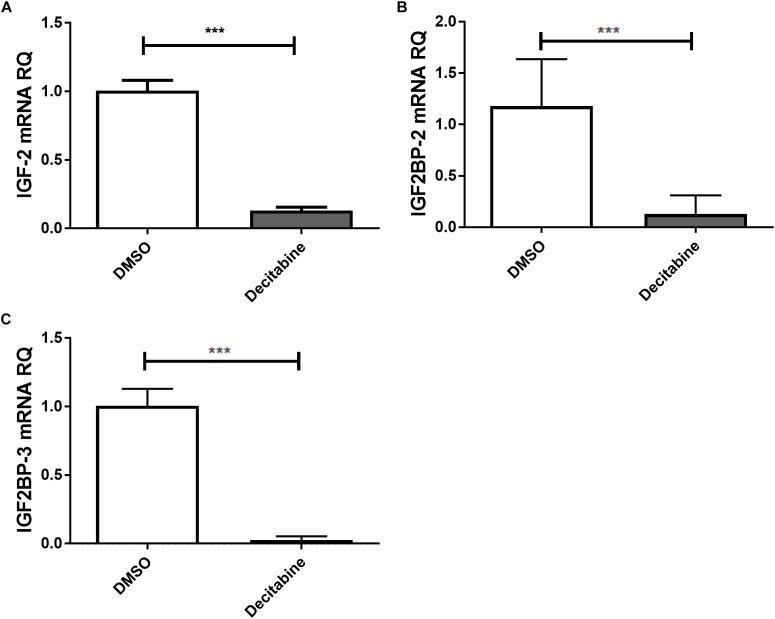
Decitabine represses IGF-II and IGF2BP-2 and 3. Huh7 cells were treated with 5 μM decitabine, a DNA methyltransferase inhibitor, for 5 days. This led to a marked suppression of IGF-II **(A)**, IGF2BP2 **(B)**, and IGF2BP3 **(C)** mRNA expression compared to untreated controls (*P* < 0.001). RQ, relative quantitation; ^∗∗∗^*P* < 0.001.

The link between epigenetically regulated miR-let-7a and the identified IGF-signaling pathway constituents is further supported by decitabine treatment experiments. In accordance with our initial hypothesis (elevated expression of miR-let-7a after inhibition of methyltransferase activity; Figure 1B), this drug is leading to a drastic reduction of IGF-II and IGF2BP-2/3 in HCC cells (Figure 4).

### MiR-let-7a Directly Targets IGF-II, IGF2BP2, and IGF2BP3 in HCC Cells

**FIGURE 5:**
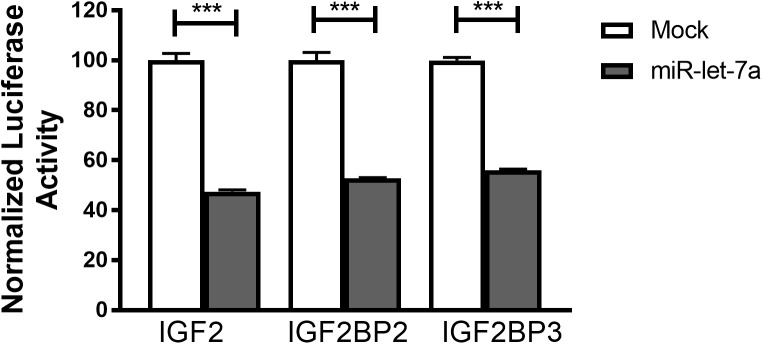
IGF-II, IGF2BP2, and IGF2BP3 are direct targets of miR-let-7a. Luciferase reporter assay shows that miR-let-7a directly targets IGF-II, IGF2BP2, and IGF2BP3, where Huh7 cells were transfected with pmiRGLO vector harboring the *in silico* predicted miR-let-7a binding site on IGF2, IGF2BP2, and IGF2BP3 3’UTR alone or co-transfected with miR-let-7a mimics. Mimics decreased the luciferase activity of IGF2 (pmirGLO_IGF2) by 52.5% (*P* < 0.0001) and IGF2BP-2 (pmirGLO_IGF2BP2) and IGF2BP-3 (pmirGLO_IGF2BP3) by a mean of 47.28% (*P* < 0.0001) and 44.03% (*P* < 0.0001), respectively; ^∗∗∗^*P* < 0.001.

To confirm that miR-let-7a is directly interacting with the identified target mRNA for IGF-II, IGF2BP-2 and IGF2BP-3, a luciferase reporter assay was performed. For that purpose, Huh7 cells were transfected with pmiRGLO vector harboring the *in silico* predicted miR-let-7a binding site on IGF-II, IGF2BP2 or IGF2BP3 3’UTR alone or co-transfected with miR-let-7a mimics. In accordance with our previous data, transfection of miR-let-7a mimics decreased the luciferase activity of IGFII by 52.5%. In addition, both analyzed IGF2BP family members were significantly reduced by 47.3% (IGF2BP2) and 44.0% (IGF2BP-3) (Figure 5). Together, these results show that miR-let-7a can directly repress IGF-II and in a direct manner, but can also repress it indirectly through targeting its binding proteins, IGF2BP-2 and -3.

## Discussion

Hepatocellular carcinoma is the fifth most common type of cancer and due to high rate of recurrence and limited chemotherapy and radiotherapy efficacy it is the second most deadly type of cancer (Ferlay et al., 2015; Mazzanti et al., 2016). HCC is characterized by dysregulation of many oncogenic signaling pathways including the insulin-like growth factor (IGF) axis. In human hepatocarcinogenesis, overexpression of the secreted ligand IGF-II is of special importance for tumor formation and activation of the relevant receptor IGF-1R correlates with poor patient prognosis (Breuhahn et al., 2006). Elevated IGF-II defines HCC patients with specific clinical features (Breuhahn et al., 2004) and supports tumor cell proliferation and migration (Nussbaum et al., 2005, 2008). In this study we now demonstrate that this oncogenic signaling axis is affected by miR-let-7a in a multi-modal manner: IGF-II as well as IGF-II-stabilizing proteins of the IGF2BP family are negatively regulated by miR-let-7a.

Up to our knowledge, our results show for the first time that the expression of miR-let-7a is downregulated in HCV-induced HCC tissues. That was further confirmed by TCGA data, where miR-let-7a shows an 0.8 fold decrease in tumor tissues compared to normal tissues (Li et al., 2014). Our finding is also in line with other findings where miR-let-7a was found to be downregulated in breast (Lu et al., 2011), lung (Brueckner et al., 2007), and pancreatic (Torrisani et al., 2009) cancers. However, a recent study has shown that miR-let-7a is downregulated in early stage HCC tissues compared to adjacent non-tumor as well as cirrhotic and chronic hepatitis tissues, but its expression in HCC was comparable to that of normal liver (Shi et al., 2017). This raises questions about potential underlying mechanisms responsible for such restricted expression in HCC. A posttranscriptional mode of miR-let-7a regulation has already been described in HCC, where the overexpressed protein Lin28B binds to the precursor miR-let-7a (pre-miR-let-7a), blocking its dicer processing and inducing its degradation (Heo et al., 2008). In this study, we tackled the question of miR-let-7a transcriptional regulation in HCC, possibly through DNA methylation. We were able to show that the identified CpG island in the *MIRLET7A3* gene is hypermethylated in HCC compared to healthy liver. The same CpG island was found to be hypermethylated in other cancer types such as epithelial ovarian cancer (Lu et al., 2007) and breast cancer (Vrba et al., 2013). However, a substantial impact of such hypermethylation on the expression of miR-let-7a was only validated in leukemia (Ko et al.). This hypermethylation was found to correlate with advanced tumor stages and poor prognostic outcomes, which sheds light on the potential significance of miRNAs coded in this region (Lu et al., 2007, 2011). Interestingly, the same CpG island was found to be hypomethylated in some lung adenocarcinoma tissues and this correlated with overexpression of miR-let-7a (Brueckner et al., 2007).

IGF2BPs constitute a family of three “oncofetal” proteins whose expression is abolished in adult tissue, yet highly re-expressed in many cancers to promote tumor invasiveness (Bell et al., 2013). Among them, IGF2BP-1 has been reported to inhibit IGF-II mRNA translation during development (Nielsen et al., 1999) while on the other hand IGF2BP-2 and -3 were previously shown to promote IGF-II mRNA translation in rhabdomyosarcoma and leukemia respectively (Liao et al., 2005; Dai et al., 2011). IGF-II signaling can be tuned by IGF2BPs, which are reported to influence the fate of IGF-II as well as IGF1 receptor (IGF1R) mRNA (Liao et al., 2011; Fawzy et al., 2016). For further investigation in HCC, we knocked down IGF2BP-2/-3, which led to a marked decrease in IGF-II expression (data not shown). This verifies the importance of IGF2BP-2 and 3 for the stability of IGF-II mRNA making it more available for the translation machinery. This finding contradicts other studies where knockdown of IGF2BP-2 and -3 was found to have no effect on IGF-II mRNA levels but rather led to decreased translation of the protein in rhabdomyosarcoma and glioblastoma cell, respectively (Dai et al., 2011; Suvasini et al., 2011). However, an IGF2BP-induced stabilization of mRNAs has been described for IGF2BP-1 which was reported to protect BTRC, C-MYC, and CD44 against miRs and the degradation machinery (Noubissi et al., 2006; Vikesaa et al., 2006; Elcheva et al., 2009). Due to the different effects of IGF2BPs on IGF-II in varying cell types, the shielding effect of IGF2BP-2 and -3 needs further validation in other cancer types.

Our findings that miR-let-7a targets both IGF2BP-2 and 3, and since IGF2BP-2 and 3 are essential for IGF-II stability, we can say that miR-let-7a can target IGF-II in HCC via two mechanisms; directly through binding to its mRNA and indirectly through targeting its stabilizers; IGF2BP-2/3. Similarly, we have previously shown that another miR-let-7 family member, namely miR-let-7i directly suppresses IGF-1R, as well as indirectly through targeting IGF2BPs in HCC (Fawzy et al., 2016), thus highlighting the crucial role played by miR-let-7 family in regulating IGF-axis members in HCC.

The epigenetic regulation of miR-let-7a and its role in regulating the identified IGF-axis members is further supported by decitabine treatment experiments. Where inhibition of DNA methyltransferases (DNMT) using decitabine induces miR-let-7a and drastically reduces IGF-II and IGF2BP-2/3 in HCC cells.

In conclusion, we provided a link between DNA methylation, miR expression and oncogenic signaling. We showed that the epigenetically repressed miR-let-7a has multilayered implications on the IGF signaling in HCC. Thus, this work enhances our understanding of the molecular mechanisms by which miR-let-7a exerts its tumor suppressor activity in HCC. This study presents miR-let-7a as a promising candidate for clinical trials with potential use in cancer therapy.

## Author Contributions

AA conceived and supervised the study. AA and KB designed the experiments. AW, NE-E, RA, and MA performed the experiments. HET co-supervised the work process. KH and GE provided liver tissues and patients clinical data. KB provided new tools and reagents. AW, NE-E, KB, and AA analyzed the data. AW wrote the manuscript. NE-E, KB, and AA made the manuscript revisions.

## Conflict of Interest Statement

The authors declare that the research was conducted in the absence of any commercial or financial relationships that could be construed as a potential conflict of interest.

## Footnotes

1 www.microrna.org

2 http://mirdb.org/

3 http://bibiserv.techfak.uni-bielefeld.de

4 http://mirtarbase.mbc.nctu.edu.tw

5 https://genome.ucsc.edu/

6 http://www.bioinformatics.org/sms2/cpg_islands.html
