# Supplementary material for: Methylation in MIRLET7A3 Gene Induces the Expression of IGF-II and Its mRNA Binding Proteins IGF2BP-2 and 3 in Hepatocellular Carcinoma
Source: Front Physiol. 2019 Jan 24;9:1918. doi: 10.3389/fphys.2018.01918 (PMC6353855; doi:10.3389/fphys.2018.01918)
Supplement: Supplementary file 1 [file Table_1.DOCX]

**Supplementary Data**

**Supplementary table 1.**

Primers used for amplification of the le-7a-3 gene used in 'Analysis of DNA methylation'

| **Primer species** | **Primer Sequence (5'--3')** |
| --- | --- |
| **Let-7a-3_Forward** | TAGAGGCCACTTCCCTCAAG |
| **Let-7a-3_Reverse** | TATCCCATAGCAGGGCAGAG |

**Supplementary table 2.**

Oligonucleotide design for wild-type (WT) 3'-UTR inserts of IGF-2, IGF2BP-2 and 3.

| **Sequence (5'--> 3')** | **Oligonucleotide species** |
| --- | --- |
| CGACTCGGCCTCTGGGAGGTTTACCTCGCCCCCAT | **IGF-2_WT_Forward** |
| CTAGATGGGGGCGAGGTAAACCTCCCAGAGGCCGAGTCGAGCT | **IGF-2_WT_Reverse** |
| CAGAAAAACACACAGAAGAAGCTACCTCAGGTGTTT | **IGF2BP-2_WT_Forward** |
| CTAGAAACACCTGAGGTAGCTTCTTCTGTGTGTTTTTCTGAGCT | **IGF2BP-2_WT_Reverse** |
| CAAACGTGCATTTTTACTCAACTACCTCAGGTATTT | **IGF2BP-3_WT_Forward** |
| CTAGAAATACCTGAGGTAGTTGAGTAAAAATGCACGTTTGAGCT | **IGF2BP-3_WT_Reverse** |

**Supplementary figure. CpG island in let-7a-3 gene.**


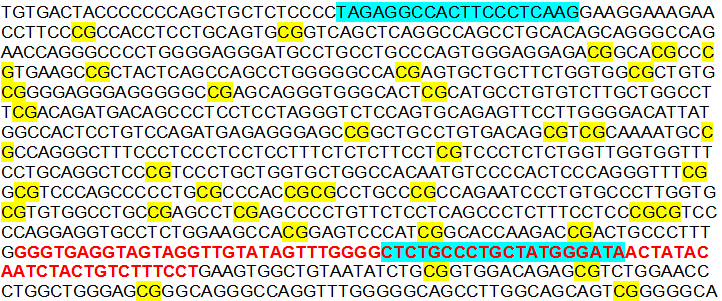


DNA sequence (5' to 3') of the CpG island identified in the let-7a-3 gene. This CpG island was identified using UCSC Genome browser as well as the online software CpG finder. The location where our primer annealing sites are, are highlighted with blue and the sequence of the precursor let-7a-3 (pre-let-7a-3) is in bold red. This CpG island resides in the genomic location 22q12.31 and stretches to ~700 bp and includes 32 CpG sites highlighted in yellow.
